# Near-Infrared Fluorescence Guidance With Indocyanine Green in Adrenal-Sparing Surgery: A Systematic Review

**DOI:** 10.7759/cureus.104597

**Published:** 2026-03-03

**Authors:** Tatiana A Kulakova, Roman A Goncharuk, Alexander V Ozherelev, Alla M Morozova, Olga G Tsygankova

**Affiliations:** 1 Surgery, Far Eastern Federal University, Vladivostok, RUS; 2 School of Medicine, Far Eastern Federal University, Vladivostok, RUS; 3 Endocrinology and Diabetes, Regional Clinical Hospital №2, Vladivostok, RUS

**Keywords:** adrenalectomy, adrenal-sparing surgery, cortical-sparing adrenalectomy, indocyanine green, near-infrared fluorescent imaging, nir/icg, partial adrenalectomy

## Abstract

Cortical-sparing adrenal surgery might require additional intraoperative navigation to optimize outcomes. Near-infrared fluorescence imaging with indocyanine green (NIR/ICG) has emerged as a promising adjunct to support this goal.

A systematic search of international and Russian databases identified clinical and experimental studies (2013-2024) evaluating NIR/ICG during adrenal surgery. Fifteen studies met the inclusion criteria. Due to data heterogeneity and limited comparative evidence, a systematic review without meta-analysis was performed.

Since the first report in 2013, subsequent studies consistently demonstrated differential fluorescence between adrenal tumors and normal tissue. NIR/ICG improved visualization of adrenal anatomy, aided margin delineation, facilitated tissue dissection, and enabled assessment of perfusion in residual adrenal tissue during cortical-sparing procedures.

NIR/ICG is a safe and promising tool for intraoperative navigation in adrenal surgery. However, further research is needed to clarify fluorescence interpretation, define correlations with tumor biology, standardize protocols, and validate its role in assessing adrenal parenchyma viability.

## Introduction and background

Organ-sparing surgical interventions (OSSs) for adrenal gland disorders allow the preservation of hormonal function and reduce the risk of chronic adrenal insufficiency and subsequent patient disability in the postoperative period.

According to studies conducted in patient groups with bilateral adrenal lesions, the group undergoing partial adrenalectomy showed a statistically significantly higher survival rate compared to those who underwent bilateral adrenalectomy requiring lifelong hormone replacement therapy, despite instances of tumor recurrence and unsuccessful preservation of endocrine function [1-4]. Chronic adrenal insufficiency developed in 23.5% of cases, and tumor recurrence following adrenal-sparing resection occurred in 3 to 13% of cases [1-3,5].

The main causes of adrenal insufficiency after partial adrenalectomy include insufficient blood supply to the remaining tissue or an inadequate amount of residual adrenal cortex required to sustain hormonal function [1,2]. Factors contributing to tumor recurrence may include tumor biology as well as undetected secondary tumors during surgery, particularly relevant for early recurrences (within the first year of follow-up) [1-3,5].

Clear radiological signs of malignancy serve as a contraindication for OSSs. The assessment of malignancy likelihood is primarily based on computed tomography (CT) imaging features [6].

Thus, organ-sparing surgeries represent a relevant surgical strategy, but achieving optimal outcomes requires enhanced intraoperative navigation to accurately determine resection margins, assess adrenal tissue perfusion, and select appropriate surgical tactics. One promising approach to addressing these challenges is near-infrared fluorescence imaging using indocyanine green dye (NIR/ICG).

This systematic review aimed to critically assess the available clinical and experimental evidence regarding the role of NIR/ICG fluorescence imaging in adrenal surgery, focusing on its impact on intraoperative visualization, perfusion assessment of residual adrenal tissue, fluorescence patterns in relation to tumor histology, and its potential influence on surgical decision-making.

## Review

Methods

Search Strategy

A systematic literature search was conducted using international (PubMed, Web of Science, EBSCO, Cochrane Library) and Russian (E-library, CyberLeninka) citation databases.

The search strategy combined controlled vocabulary (MeSH terms, where applicable) and free-text keywords. The following search string was used and adapted for each database:

(("adrenalectomy" OR "partial adrenalectomy" OR "cortical-sparing adrenalectomy" OR "adrenal-sparing surgery") 

AND ("indocyanine green" OR "ICG" OR "near-infrared fluorescence" OR "NIR/ICG"))

The search was limited to studies published between January 2013 and December 2024 in English and Russian. In addition, the reference lists of included articles were manually screened to identify potentially relevant studies not captured in the initial search.

Inclusion criteria: Clinical and experimental studies published between 2013 and 2024 in Russian and English that examined the use of indocyanine green dye for near-infrared fluorescence imaging (NIR/ICG) during adrenal surgeries. Studies analyzing outcomes of both adrenal-sparing and radical surgeries, as well as those conducted on experimental models, were included. Evaluated parameters included operative time, blood loss, visualization, fluorescence dependency on tumor morphology, immunohistochemical and molecular-genetic structure, complications, and postoperative outcomes.

Exclusion criteria: Studies concerning tumors of other organs, non-peer-reviewed publications, and works lacking data on the results of NIR/ICG in surgery were excluded. Also excluded were in vitro studies, reviews, meta-analyses, editorials, letters to the editor, and articles published in languages other than Russian and English.

Data Extraction

Two reviewers independently screened titles and abstracts for eligibility. Full-text articles were subsequently assessed according to predefined inclusion and exclusion criteria. Data were extracted using a standardized data collection form that included study design, patient population, surgical approach, intraoperative parameters, fluorescence characteristics, histological findings, recurrence rates, and postoperative hormonal outcomes. Disagreements between reviewers were resolved through discussion and consensus.

**Figure 1:**
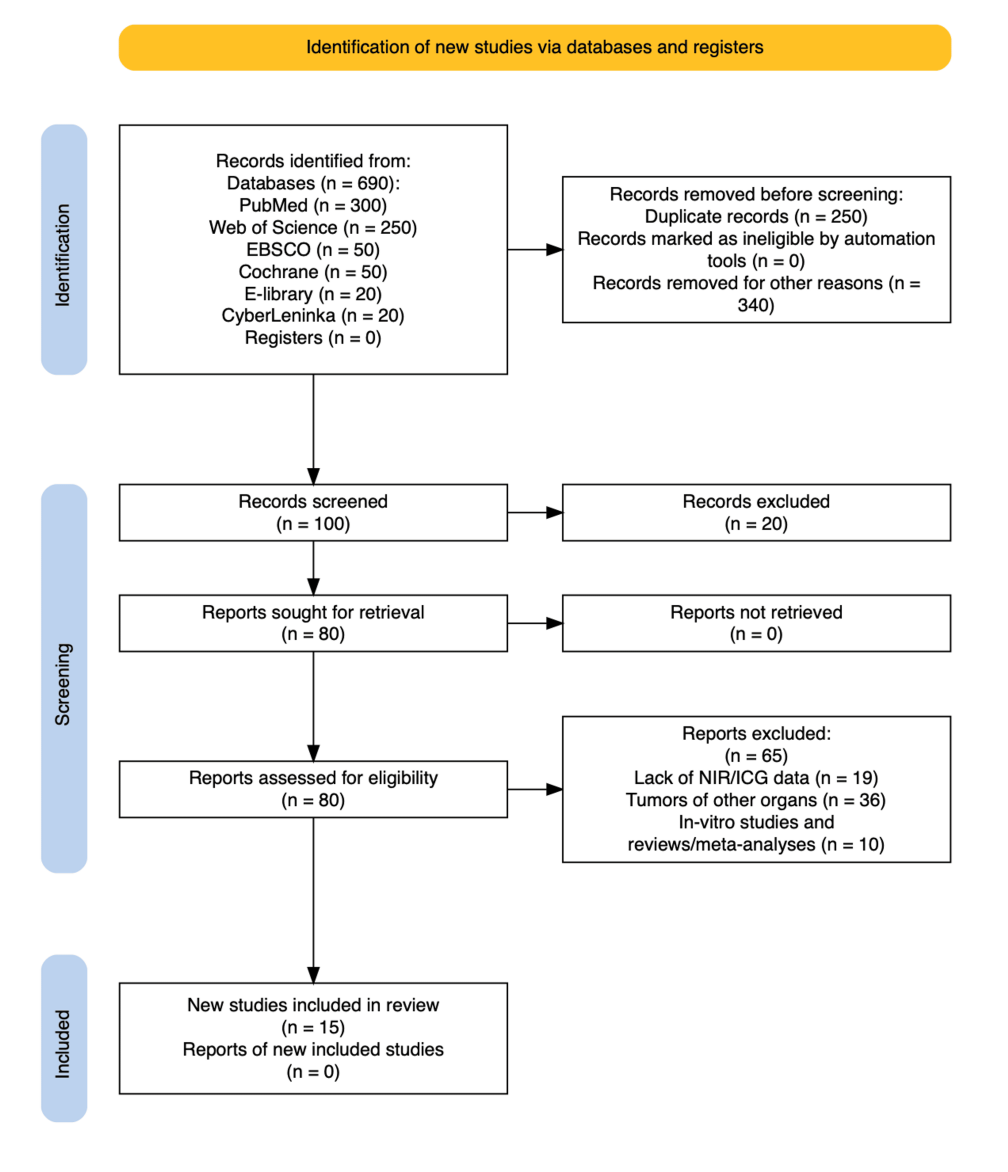
PRISMA Flow Diagram PRISMA: Preferred Reporting Items for Systematic reviews and Meta-Analyses

The publication selection process followed Preferred Reporting Items for Systematic reviews and Meta-Analyses (PRISMA) guidelines and is illustrated using a PRISMA flow diagram [7], shown in Figure 1.

Results

Indocyanine green (ICG) is a tricarbocyanine dye that fluoresces under near-infrared light at approximately 800 nm. As ICG lacks tumor specificity, its fluorescence patterns reflect vascular and tissue characteristics rather than direct molecular binding [8-11].

Overview of Included Studies

In total, 15 studies were included in the literature review. They covered data on operative time, surgical approach, intraoperative blood loss, complications, histological outcomes, correlation with fluorescence patterns, CT density, and its relationship to ICG uptake, recurrence rates, and postoperative hormonal status. Due to the insufficient number of publications with control groups, a meta-analysis was not feasible; therefore, a systematic review was conducted.

Given the predominantly observational nature of the available evidence, the methodological quality of the included studies was systematically evaluated. Clinical studies were assessed using the Newcastle-Ottawa scale (NOS), while preclinical animal studies were evaluated according to the ARRIVE 2.0 guidelines. The results of this quality assessment are summarized in Table 1.

**Table 1:** Methodological Quality Assessment of Included Studies Using the Newcastle–Ottawa Scale (NOS) for Clinical Studies and ARRIVE 2.0 Guidelines for Preclinical Studies

No.	Study	Quality Assessment Scale	Score	Comments
1	Manny et al., 2013 [12]	NOS	6/9	Small pilot study; no comparative analysis
2	Tuncel et al., 2019 [13]	Newcastle-Ottawa Scale (NOS)	6/9	No control group; pilot study
3	Balescu et al., 2019 [14]	NOS	6/9	No comparison group; descriptive study design
4	Lerchenberger et al., 2020 [15]	NOS	6/9	Limited sample size; no comparative analysis
5	Manuel et al., 2024 [16]	NOS	8/9	Lack of a control group; however, the prospective protocol was well-documented
6	DeLong et al., 2015 [17]	NOS	6/9	Very limited sample size; no control group
7	Arora et al., 2018 [18]	NOS	6/9	Retrospective case series; no control group
8	Sound et al., 2016 [19]	NOS	6/9	No control group; small sample size
9	Colvin et al., 2016 [20]	NOS	6/9	Pilot study; no comparison group
10	Kahramangil et al., 2018 [21]	NOS	8/9	Well-controlled variables; absence of a control group
11	Seeliger et al., 2019 [22]	ARRIVE 2.0	17/20	Preclinical animal study. The study demonstrated a high level of reporting and transparency; limitations included lack of randomization, blinding, and data availability
12	Berber et al., 2023 [23]	NOS	7/9	Absence of a control group and long-term follow-up
13	Berber et al., 2024 [24]	NOS	7/9	Absence of a control group and long-term follow-up
14	Palomba et al., 2022 [25]	NOS	9/9	Propensity score-matched study; comprehensive assessment
15	Liu et al., 2024 [26]	NOS	7/9	Includes a comparison group; however, lacks multivariate analysis and long-term follow-up

Early Clinical Feasibility and Intraoperative Navigation

The first clinical application of NIR/ICG in adrenal surgery was reported by Manny et al. (2013). Heterogeneity in fluorescence between normal and tumor tissues was identified, with all tumors (pheochromocytoma, lipoadenoma, and follicular lymphoid hyperplasia) appearing hypofluorescent. This contrast helped delineate the resection margin and achieve R0 resection. The study confirmed the safety of using NIR/ICG in adrenal surgery [12].

Subsequent authors (Tuncel et al., Balescu et al., Lerchenberger et al., Juan Manuel et al., DeLong et al., Arora et al., Sound et al., Colvin et al.) described early experiences with this method. They reported differential ICG uptake between adrenal and tumor tissues. The technique was primarily used to visualize the adrenal gland within surrounding tissues, assisting in dissection and vascular identification. In all cases, NIR/ICG supported intraoperative navigation, although Arora et al. and Sound et al. noted reduced effectiveness on the right side due to hepatic fluorescence [13-20]. In 2019, Lerchenberger et al. performed adrenal-sparing surgery in three patients with bilateral adrenal lesions. They noted improved visualization of tumor margins during pheochromocytoma resection. However, due to the small sample size, the study was descriptive and lacked statistical analysis [15]. In the study by Manuel et al., the use of NIR/ICG influenced surgical decision-making in favor of adrenalectomy [16].

Fluorescence Patterns in Relation to Tumor Histology

In a 2018 study by Kahramangil et al. involving 100 patients, the authors found that ICG uptake intensity correlated with the histological type of tumor. Tumors of cortical origin exhibited intense fluorescence compared to normal parenchyma, while medullary tumors (pheochromocytomas) appeared less fluorescent. The study focused on total adrenalectomy and did not assess adrenal-sparing procedures in relation to tumor histology [21]. Seeliger et al. also reported enhanced identification and simplified dissection of the adrenal gland but did not evaluate differences in fluorescence among tumor types [22].

In the first study by Berber et al., fluorescence of pheochromocytomas was analyzed in 46 cases. A statistically significant association between tumor fluorescence and histology was observed. The PASS score and other factors did not influence fluorescence [23]. In November 2024, Berber et al. published a study evaluating fluorescence patterns and histological findings in 197 patients undergoing robotic adrenalectomy between 2014 and 2023. Intraoperative fluorescence was subjectively assessed and classified as “fluorescent” or “non-fluorescent,” while histology categorized tumors as benign or malignant. The technique demonstrated high sensitivity (87%) and positive predictive value (95.2%), but low specificity (38.5%) and negative predictive value (17.2%) for distinguishing benign from malignant tumors. These findings suggest that NIR/ICG may assist in identifying benign adrenocortical tumors; however, its ability to exclude malignancy remains limited [24].

Comparative Clinical Studies

To improve surgical planning and intraoperative decision-making, Palomba et al. compared outcomes of laparoscopic adrenalectomy in two groups: (i) with preoperative CT-based 3D reconstruction and NIR/ICG visualization and (ii) standard protocol without additional visualization. The method proved safe and led to statistically significant reductions in operative time and blood loss. The approach enhanced navigation (e.g., resection margins, vascular supply, anatomical variations) [25].

Another comparative study by Liu et al. (2024) assessed retroperitoneoscopic adrenalectomy with and without ICG. Statistically significant differences were noted in duration of surgery, tumor detection time, blood loss, and pain VAS, all favoring the ICG group. No other significant differences were observed [26].

Assessment of Residual Adrenal Perfusion in Cortical-Sparing Surgery

Additionally, NIR/ICG was used to assess the viability of the remaining adrenal segment. Kahramangil et al., Balescu et al., and Manuel et al. evaluated perfusion of residual tissue by administering additional ICG doses [15,16,21].

In an animal study by Seeliger et al., significant differences in fluorescence were observed between ischemic and intact adrenal tissue during resection. Fluorescence of adrenal segments was compared with that of the ipsilateral healthy kidney, but differences between cortical and medullary adrenal tissues were not assessed [22].

Regarding dissection impact on tissue viability, venous anastomoses in the caudal adrenal capsule were observed. These may serve as alternate venous drainage pathways, allowing for central adrenal vein division without compromising the function of the remaining gland. This was confirmed by persistent fluorescence following central vein division [21,22].

Evidence From Russian Literature

In Russian literature, there are no original studies on ICG-assisted adrenal-sparing surgery, with only review articles based on international research [27,28].

Discussion

Among the original studies on the use of NIR/ICG in adrenal surgery, only two included comparison groups [25,26]. However, due to heterogeneity of the data, a comparative analysis could not be performed, and no statistically significant conclusions could be drawn. Therefore, the majority of existing literature consists of case series.

The available data indicate that intraoperative fluorescence navigation enables the visualization of the adrenal gland within surrounding tissues and identification of major supplying vessels, thereby facilitating dissection [12-26]. When performing adrenal-sparing procedures, it also allows for the assessment of blood supply and viability of the remaining parenchyma [15,16,21,22].

In clinical studies, authors noted that fluorescence depends on the histological structure of the tumor. Cortical tumors exhibit more intense fluorescence compared to normal parenchyma, while medullary tumors (pheochromocytomas) appear less fluorescent [12-16,18-21,23-25]. The degree of pheochromocytoma fluorescence might depend on tumor size [23]. These features assist in tumor margin identification and have potential as an additional intraoperative criterion for determining surgical strategy.

All fluorescence evaluations in clinical research were subjective and lacked standardized scales or gradation criteria. None of the studies proposed an objective scoring system. Moreover, the persistence of fluorescence for up to 30 minutes after initial ICG injection raises the question of whether this could impact the assessment of residual adrenal viability upon repeated dosing.

The combination of CT-based 3D reconstruction and NIR/ICG was applied to improve operative field navigation [25]. However, no authors evaluated the correlation between NIR/ICG and CT imaging features.

It remains unclear whether NIR/ICG alone is sufficient for addressing the main challenges of adrenal-sparing surgery or whether it requires integration with other diagnostic and intraoperative navigation methods.

No studies that analyzed the correlation between fluorescence patterns and immunohistochemical or molecular-genetic characteristics of adrenal tumors were identified.

## Conclusions

This systematic review demonstrates that NIR/ICG fluorescence imaging represents a safe and valuable intraoperative adjunct in adrenal surgery, enhancing visualization of adrenal anatomy and facilitating perfusion assessment of residual adrenal tissue during cortical-sparing procedures. Available evidence suggests an association between fluorescence patterns and tumor histology; however, current assessments remain subjective and lack standardized grading systems. To further define the clinical role of NIR/ICG, future research should prioritize prospective comparative studies and the development of objective fluorescence evaluation criteria, with correlation to postoperative hormonal function and oncologic outcomes. Establishing such standardized approaches may enhance not only the surgical technique but also intraoperative decision-making and overall treatment strategies for patients with adrenal tumors.
